# The Amelioration of N-Acetyl-p-Benzoquinone Imine Toxicity by Ginsenoside Rg3: The Role of Nrf2-Mediated Detoxification and Mrp1/Mrp3 Transports

**DOI:** 10.1155/2013/957947

**Published:** 2013-05-14

**Authors:** Sang Il Gum, Min Kyung Cho

**Affiliations:** Department of Pharmacology, College of Oriental Medicine, Dongguk University, Kyungju 780-714, Republic of Korea

## Abstract

Previously, we found that Korean red ginseng suppressed acetaminophen (APAP)-induced hepatotoxicity via alteration of its metabolic profile involving GSTA2 induction and that ginsenoside Rg3 was a major component of this gene induction. In the present study, therefore, we assessed the protective effect of Rg3 against N-acetyl-p-benzoquinone imine (NAPQI), a toxic metabolic intermediate of APAP. Excess NAPQI resulted in GSH depletion with increases in the ALT and AST activities in H4IIE cells. Rg3 pretreatment reversed GSH depletion by NAPQI. Rg3 resulted in increased mRNA levels of the catalytic and modulatory subunit of glutamate cysteine ligase (GCL), the rate-limiting steps in GSH synthesis and subsequently increased GSH content. Rg3 increased levels of nuclear Nrf2, an essential transcriptional factor of these genes. The knockdown or knockout of the Nrf2 gene abrogated the inductions of mRNA and protein by Rg3. Abolishment of the reversal of GSH depletion by Rg3 against NAPQI was observed in Nrf2-deficient cells. Rg3 induced multidrug resistance-associated protein (Mrp) 1 and Mrp3 mRNA levels, but not in Nrf2-deficient cells. Taken together, these results demonstrate that Rg3 is efficacious in protecting hepatocytes against NAPQI insult, due to GSH repletion and coordinated gene regulations of GSH synthesis and Mrp family genes by Nrf2.

## 1. Introduction

Ginseng, one of the most commonly used herbal medicines, has been reported to be adaptogenic in the endocrine, immune, cardiovascular, and central nervous systems [1, 2]. Previously, we reported that ginseng has the potential to protect against benzo[*α*]pyrene and acetaminophen (APAP) [3, 4]. The ginseng saponins, referred to as ginsenosides, play a key role for most of the physiological and pharmacological activities of ginseng [5]. In angiogenesis, the ginsenosides Rb1 and Rg1 showed opposing activities [6]. Ginsenoside Rg3 is responsible for various pharmacological actions of red ginseng, including antitumor activity, antihyperglycemic activity, and cardio protection [7–9]. We demonstrated that Rg3, but not Rb1, Rc, or Rg1, accounted for the significant induction of glutathione S-transferase A2, which may facilitate catalysis of glutathione conjugation with APAP metabolites [4]. 

Hepatotoxicity induced by excess APAP, a widely used analgesic and antipyretic drug, is the most common cause of death by acute liver failure [10]. N-acetyl-p-benzoquinone imine (NAPQI), a toxic metabolic intermediate, is a major cause of hepatic necrosis as a result of high doses of APAP [10]. The reactive electrophile is subsequently converted to an inactive product through additional metabolic processes involving phase II detoxifying enzymes, which eliminate toxic metabolites [11]. However, overdose of APAP rapidly depletes hepatic glutathione (GSH), and the remaining NAPQI results in hepatocellular necrosis. N-acetylcysteine, a precursor of GSH, is the primary therapeutic treatment for APAP overdose [12]. However, a low therapeutic window and critical timing of N-acetylcysteine therapy is a limiting factor for treatment of APAP poisoning because the GSH level alone is not enough to protect against it [12, 13].

Antioxidant defense systems are composed of GSH and its synthesis, phase II detoxifying enzyme, and reactive oxygen species inactivating enzymes, which play key roles in protecting cells upon oxidative damage including that caused by NAPQI [14]. Glutamate cysteine ligase (GCL) is the rate-limiting enzyme for de novo GSH synthesis and comprises heterodimeric proteins formed of a catalytic and a modulatory subunit (GCLC and GCLM, resp.) [15]. Phase II drug-metabolizing enzymes, such as glutathione S-transferase and NAD(P)H:quinone oxidoreductase 1 (NQO1), serve to decrease the damage caused by reactive intermediates. The transcription factor nuclear factor-erythroid 2-related factor 2 (Nrf2) that binds to the antioxidant response element (ARE) exerts an essential effect on the transcriptional activation of this gene induction [16].

In addition to the GSH pool and hepatic detoxification, Nrf2 stimulates hepatic multidrug resistance-associated protein (Mrp) transport, which exerts efflux of xenobiotics and metabolites instead of their accumulation [17]. Mrp2 transports conjugates of glucuronate, sulfate, and GSH into the bile (from hepatocyte) or urine (from renal proximal tubular cells), whereas Mrp3 and Mrp4 in the basolateral membrane transport these into the bloodstream towards renal excretion [17, 18]. High levels of APAP glucuronide in the plasma resulting from hepatic Mrp3 induction are excreted in the kidney, which suppresses APAP toxicity [14, 19, 20]. The dysregulation of ARE in the protective antioxidant defense system and transporters may be attributed to high sensitivity to APAP hepatotoxicity in the Nrf2-knockout mice [11, 14]. The coordination of detoxification and transport pathways by Nrf2 may enhance action in the mitigation of cellular injury.

In the present study, we demonstrated for the first time that Rg3 attenuated NAPQI-induced toxicity, which is attributed to GSH repletion, as well as induction of cellular defense genes including GCLC, GCLM, and basolateral Mrp transports via Nrf2 activation. We conclude that the beneficial effects of the ginsenoside Rg3 may contribute to detoxification and excretion of NAPQI metabolites suggesting that Rg3 should be considered as a potential hepatoprotective agent against NAPQI-induced damage. 

## 2. Materials and Methods

### 2.1. Reagents

**Figure 1:**
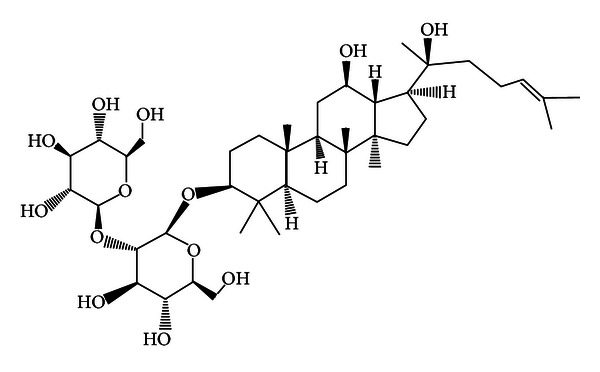
The structure of 20(S)-ginsenoside Rg3.

Anti-GCLC, Anti-GCLM, Anti-LaminA, and Anti-Nrf2 antibodies were obtained from Santa Cruz Biotechnology (Santa Cruz, CA, USA). Horseradish peroxidase-conjugated goat anti-rabbit and rabbit anti-goat IgGs were purchased from Zymed Laboratories (San Francisco, CA, USA). NAPQI, 20(S)-ginsenoside Rg3 (Figure 1), and other reagents in the molecular studies were acquired from Sigma Chemical (St. Louis, MO, USA). Nrf2 small interfering RNA (siRNA) and nontargeting scrambled RNA (control siRNA) were obtained from Bioneer Co. (Daejeon, Republic of Korea). Murine embryonic fibroblasts (MEFs) from wild-type and Nrf2-disrupted mice were kindly received by M. K. Kwak, Catholic University, Gyeonggi, Republic of Korea [21].

### 2.2. Cell Culture

H4IIE cells (ATCC CRL-1548), a rat hepatocyte-derived cell line, were obtained from American Type Culture Collection (Rockville, MD, USA). The cells were maintained in Dulbecco's modified Eagle's medium containing 10% fetal calf serum, 50 units/mL penicillin, and 50 *μ*g/mL streptomycin at 37°C in a humidified atmosphere with 5% CO_2_. MEFs from wild-type and Nrf2-disrupted mice were maintained in Iscove's modified Dulbecco's medium (HyClone, Logan, UT, USA) containing 10% fetal bovine serum, 4 mM L-glutamine, Hepes and 50 units/mL penicillin, and 50 *μ*g/mL streptomycin at 37°C.

### 2.3. Determination of Alanine Aminotransferase (ALT) and Aspartate Aminotransferase (AST) Levels

The activities of ALT and AST, as markers of liver injury, were measured in the media using cobas 6000 chemistry analyzer (Roche, Mannheim, Germany).

### 2.4. Glutathione Assay

The GSH levels of cell homogenates were determined by using assay kit GSH BIOXYTECH GSH-400 (Oxis International Inc., Portland, OR, USA) and operated in accordance with the instruction. 

### 2.5. Reverse Transcription-Polymerase Chain Reaction (RT-PCR)

Total RNA was isolated from cells with TRIzol (Invitrogen, Carlsbad, CA, USA). RT-PCR amplification was conducted as previously described [22]. The number of amplification cycles was empirically determined for each primer pair to identify the logarithmic phase. The selective primer sets for GCLC, GCLM, Mrp1, Mrp2, Mrp3, Mrp4, and *β*-actin were designed to have a *T*_*m*_ of approximately 55°C and a GC content of ~50%; BLAST searches were used to confirm the specificity of the selected nucleotide sequences. Band intensities of the amplified DNAs were compared after visualization on a UV transilluminator.

### 2.6. Immunoblot Analysis

Sodium dodecyl sulfate-polyacrylamide gel electrophoresis and immunoblot analyses were performed in lysates and nuclear protein from cells according to previously published procedures [22, 23]. The resulting image was developed using the ECL chemiluminescence detection kit (Amersham Biosciences, Amersham, UK). Equal loading of proteins was verified by *β*-actin or lamin A immunoblotting. Changes in the protein levels were determined using a Gel-Pro Analyzer (Media Cybernetics Inc, Bethesda, MD, USA). 

### 2.7. siRNA Knockdown

H4IIE cells were transiently transfected with siRNA directed against rat Nrf2 or control siRNA (20 nM) using Lipofectamine 2000 (Invitrogen, Carlsbad, CA, USA) according to the manufacturer's instruction. The Nrf2 knockdown was confirmed in the protein samples.

### 2.8. Statistical Analysis

One-way analysis of variance (ANOVA) procedures were used to assess significant differences among treatment groups. For each significant effect of treatment, the Newman-Keuls test was used for comparisons of multiple group means. All statistical tests were two sided.

## 3. Results

### 3.1. The Protective Effect of Rg3 against NAPQI

**Figure 2:**
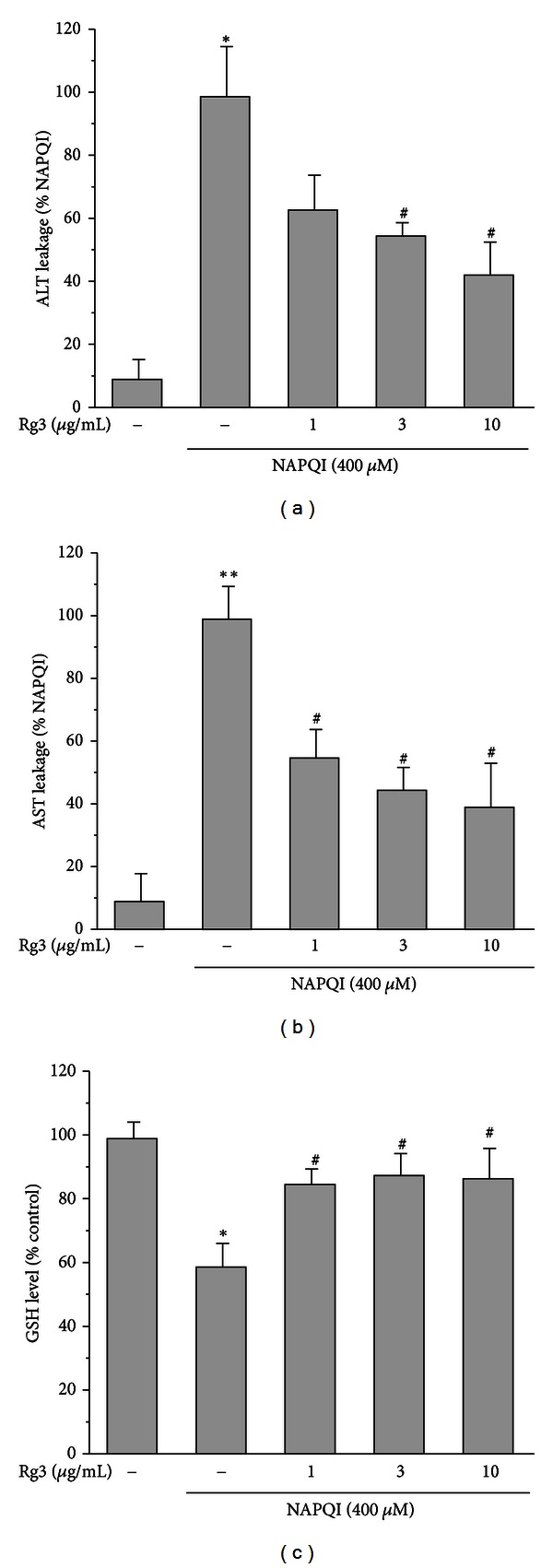
The protective effect of ginsenoside Rg3 on the NAPQI-induced toxicity in H4IIE cells. The cells were treated with the indicated concentrations of Rg3 or vehicle for 24 h. Then, the cells were incubated with NAPQI (400 *μ*M) for 6 h. (a) ALT and (b) AST levels were monitored in the media to determine cytotoxicity. (c) GSH levels were determined in cell homogenates after exposure of the cells to NAPQI (400 *μ*M) for 2 h after the cells had been pretreated or not pretreated with Rg3 for 24 h. All experiments were performed in quadruplicate. Each value represents the mean ± S.E. (**P < 0.01, *P < 0.05, significantly different from control; ^#^ *P* < 0.05, significantly different from NAPQI).

A high dose of NAPQI causes hepatic toxicity [4, 10]. We found increases in ALT and AST levels as a result of treatment with NAPQI at a dose greater than 200 *μ*M for 6 h in H4IIE, hepatocyte-derived cells (data not shown). To examine whether Rg3 is protective against NAPQI-induced toxicity, cells were pretreated with Rg3 for 24 h prior to treatment with NAPQI (400 *μ*M) for 6 h. The ALT or AST leakage in the supernatant of cells treated with NAPQI was suppressed in a dose-dependent manner by Rg3 treatment (Figures 2(a) and 2(b)). Excess NAPQI rapidly depletes GSH, and the remaining NAPQI results in hepatic necrosis [10]. Additionally, we assessed the GSH level after the administration of a high dose of NAPQI for 2 h with or without Rg3. The suppression of GSH levels by NAPQI was 60% of the control level (Figure 2(c)). In contrast, the cells pretreated with 1–10 *μ*g/mL Rg3 reversed the NAPQI-mediated GSH suppression. These results suggest that increasing GSH content by Rg3 treatment may be a beneficial mechanism for protection against NAPQI.

### 3.2. The Effects of Rg3 on the GSH Synthesis Gene Expression and GSH Content

**Figure 3:**
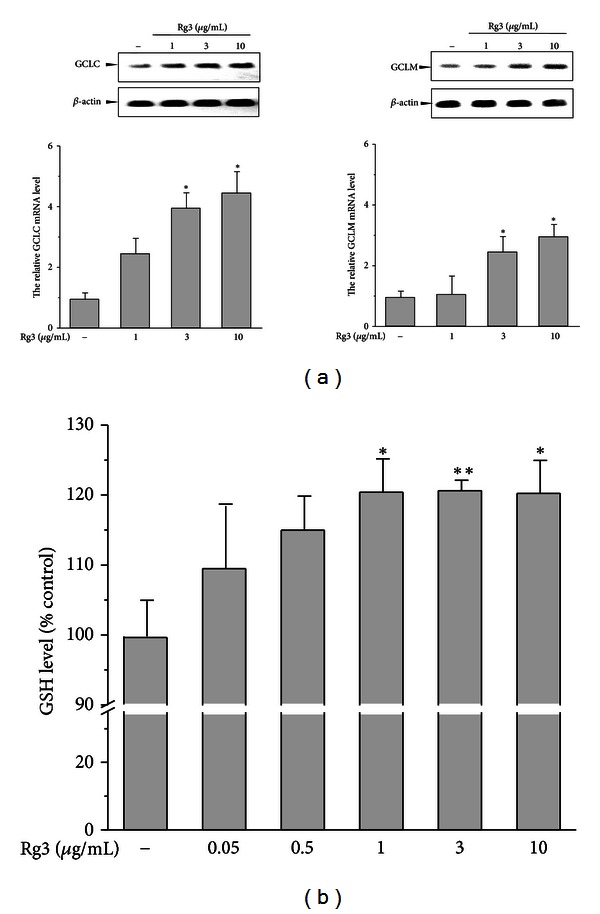
Effects of Rg3 on the GCLC and GCLM gene expression and GSH content. (a) The cells were treated with Rg3 (1–10 *μ*g/mL) for 12 h. The representative blots of GCLC and GCLM mRNA were assessed by RT-PCR. Band intensities of gene products were quantified and normalized to *β*-actin. (b) The GSH level was determined in the cell homogenates with the indicated dose of Rg3 for 24 h. The value in the graph represents the mean ± S.E. with at least three separate experiments (*P < 0.05, **P < 0.01, significantly different from control).

GCL which is the rate limiting step in GSH synthesis, controls the biosynthesis of reduced GSH form [24]. We examined GCLC, and GCLM gene expression to address the role of GSH synthesis in GSH production. Treatment of Rg3 at a dose of 3–10 *μ*g/mL significantly increased GCLC and GCLM mRNA levels, with maximal inductions of 4.5-fold and 3-fold at 12 h, respectively (Figure 3(a)). We further evaluated whether these gene inductions lead to an increase in GSH levels. As shown in Figure 3(b), the GSH level was increased in a dose-dependent manner by Rg3, showing a saturated pattern at a dose of 1 *μ*g/mL. These results show that a significant increase in GSH content is accompanied by gene inductions of GCLC and GCLM by Rg3.

### 3.3. Rg3-Mediated Nuclear Translocation of Nrf2

**Figure 4:**
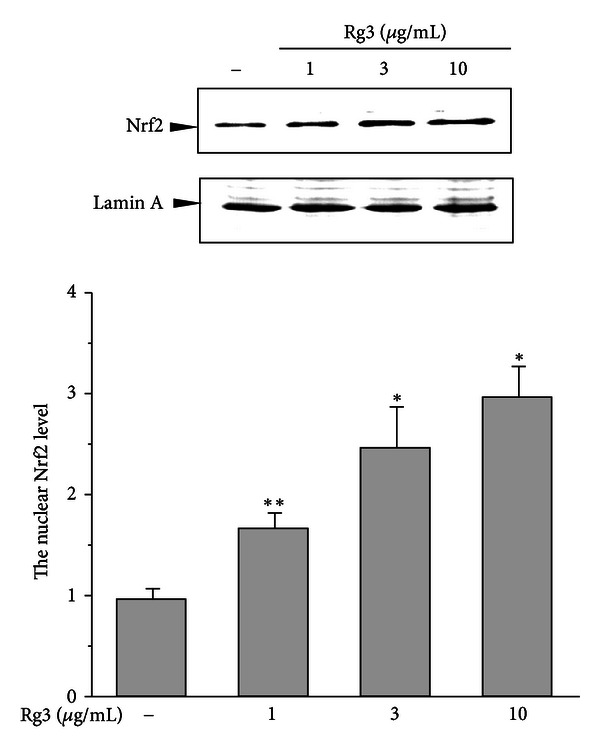
Nuclear Nrf2 translocation by Rg3. Nuclear fractions were prepared from H4IIE cells incubated with Rg3 (1–10 *μ*g/mL) for 8 h. Nuclear Nrf2 and Lamin A were immunoblotted with their respective antibodies. Lamin A was used as the loading control. The graph of multiple analyses of repeated experiments was assessed by scanning densitometry of Nrf2. Each value represents the mean ± S.E. (*P < 0.05, **P < 0.01, significantly different from control).

The nuclear Nrf2 plays a key role in the transactivation of GCLC and GCLM [11]. To determine whether Rg3 induces Nrf2 nuclear translocation, we evaluated Nrf2 expression in the nucleus of cells treated with 1–10 *μ*g/mL Rg3. The nuclear translocation of Nrf2 after Rg3 treatment was significantly increased in a dose-dependent manner at 8 h (Figure 4). This data suggests that Nrf2 activation by Rg3 might be associated with the regulations of GCLC and GCLM.

### 3.4. The Role of Nrf2 in the GCLC and GCLM Induction by Rg3

**Figure 5:**
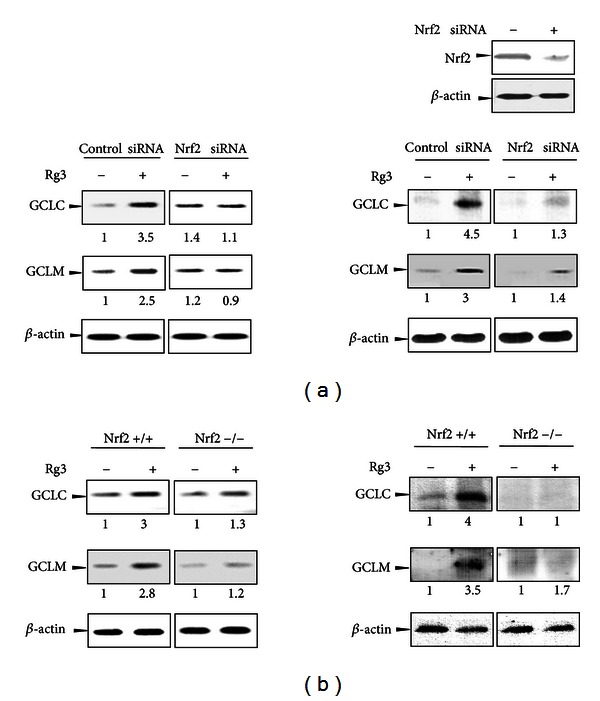
The Nrf2-mediated GCL gene expression by Rg3. (a) H4IIE cells were transiently transfected with siRNA directed against Nrf2 or nontargeted siRNA and subsequently treated with Rg3 (3 *μ*g/mL) or vehicle for 12 h. The representative blots of mRNA after Rg3 treatment were assessed by RT-PCR (left). To confirm gene knockdown, the Nrf2 levels 48 h after siRNA transfection were determined in the cell lysates by western blotting. GCLC and GCLM protein expression in the total cell lysates were detected in cells stimulated with Rg3 (3 *μ*g/mL) for 24 h (right). *β*-actin was used as the loading control. (b) MEFs from wild-type (Nrf2+/+) or Nrf2-deficient mice (Nrf2−/−) were treated with Rg3 (3 *μ*g/mL) or vehicle for 12 h. The mRNA levels (left) and protein expression (right) for the genes were analyzed as described above (a). All experiments were performed using independent mRNA and lysate samples. Each value represents the average with at least three separate experiments.

To assess whether the activation of Nrf2 by Rg3 is critical for gene induction, we performed Nrf2-gene knockdown with Nrf2 targeting siRNA in H4IIE cells. Treatment with Rg3 (3 *μ*g/mL) at 12 h increased GCLC and GCLM mRNA levels in the control group but not in Nrf2-specific siRNA transfected cells (Figure 5(a), left). To determine whether the changes in the mRNA levels were accompanied by changes in the protein expressions, we performed a western blot analysis of cell lysates treated with Rg3 (3 *μ*g/mL) for 24 h in both cell groups. Rg3 significantly elevated the GCLC and GCLM protein expression by 4.5-fold and 3-fold, respectively, which was consistent with mRNA levels (Figure 5(a), right). In contrast, the induction of GCLC and GCLM expression by Rg3 was attenuated in Nrf2-gene knockdown cells (Figure 5(a), right). The baseline control expression of GCLC and GCLM was very low in Nrf2-siRNA transfected cells. The gene knockdown by Nrf2 siRNA at 48 h was confirmed using western blotting (Figure 5(a), right up). Next, we analyzed gene expressions that were induced by Rg3 in the Nrf2−/− cells to confirm the role of Nrf2. Rg3 increased GCLC and GCLM mRNA levels by ~3-fold in the control group, whereas this induction was attenuated in the Nrf2-knockout group (Figure 5(a), left). Rg3 increased GCLC and GCLM protein expression by 4-fold and 3.5-fold, respectively, in the wild-type MEF but not in the Nrf2−/− MEF. This difference in gene expression in response to Rg3 between control and Nrf2 deficiency supports the idea that Nrf2 activation is critical for gene inductions by Rg3, which leads to mitigated NAPQI toxicity.

### 3.5. Attenuation of GSH Reversal by Rg3 against NAPQI in the Nrf2-Gene Knockdown

**Figure 6:**
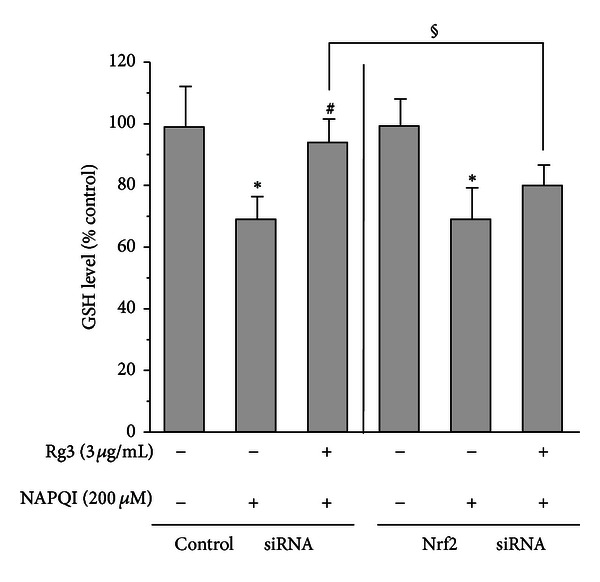
The GSH levels after NAPQI treatment with Rg3 in Nrf2-deficient cells. H4IIE cells were transiently transfected with siRNA directed against Nrf2 or control siRNA and subsequently treated with Rg3 (3 *μ*g/mL) or vehicle for 24 h. Then, cells were incubated with NAPQI (200 *μ*M) for 2 h. The GSH level was determined in the cell homogenates. All experiments were performed in quadruplicate. Each value represents the mean ± S.E. (*P < 0.05, significantly different from each control; ^#^ *P* < 0.05, significantly different from each NAPQI; ^§^ *P* < 0.05, significantly different between the 2 groups).

Reduced GSH repletion is necessary for cell survival under conditions of GSH depletion and cytotoxicity [24]. To prove the role of Nrf2 in the reversal of GSH by Rg3 in response to NAPQI, we determined GSH levels after NAPQI exposure with or without Rg3 (3 *μ*g/mL) in gene knockdown cells using Nrf2-targeting siRNA. Treatment with NAPQI (200 *μ*M) at 2 h readily suppressed GSH levels, and this effect was significantly reversed by Rg3 pretreatment in the control group (Figure 6). However, Rg3 failed to reverse NAPQI-mediated GSH inhibition in the Nrf2-knockdown group. These findings prove that Nrf2 is required for the Rg3-mediated GSH regulation in hepatocytes in response to NAPQI toxicity.

### 3.6. The Differential Gene Expressions of Mrp Family Members Induced by Rg3

**Figure 7:**
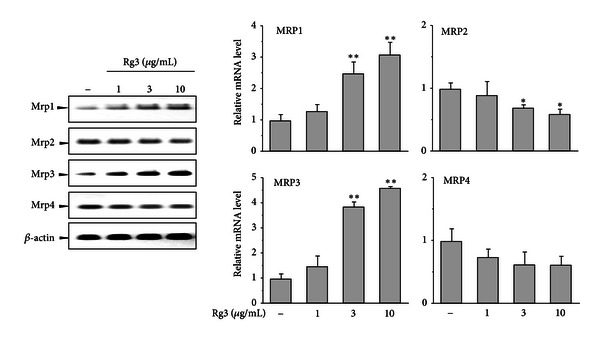
The gene expressions of Mrp family members by Rg3. Quantitative RT-PCR was performed with total RNA fractions from cells treated with Rg3 at the indicated dose for 12 h. The loading of RNA in each lane was normalized with *β*-actin as the loading control. The graphs were obtained from 3 separate experiments. Each value represents the mean ± S.E. (**P < 0.01, *P < 0.05, significantly different from control).

GCL and Mrp coexpression in many systems suggests that these two genes are coordinately regulated [18]. Mrp family transport expression determines the efflux of APAP metabolites, resulting in alteration of susceptibility to APAP hepatotoxicity [14, 18]. Hence, we measured the Mrp family mRNA levels induced by Rg3 at the indicated doses using quantitative RT-PCR. Interestingly, Rg3 differentially regulated Mrp mRNA levels (Figure 7). Mrp1 mRNA levels were significantly increased in a dose-dependent manner after Rg3 treatment, showing a maximal induction of 3-fold at 12 h, although the basal expression of Mrp1 in the liver is very low compared to the other isozymes. Unexpectedly, Rg3 caused dose-dependent suppression of Mrp2 mRNA levels. Rg3 at the dose of 10 *μ*g/mL significantly increased the levels of Mrp3 mRNA by 4.5-fold, whereas the level of Mrp4 mRNA was not significantly altered. When we considered these effects on Mrp gene expression, it is possible that Rg3 will cause NAPQI metabolite efflux into the basolateral membrane and inhibited transport into bile.

### 3.7. Nrf2 Knockdown Blocks the Alteration of Mrp Family Transporter Gene Expressions by Rg3

**Figure 8:**
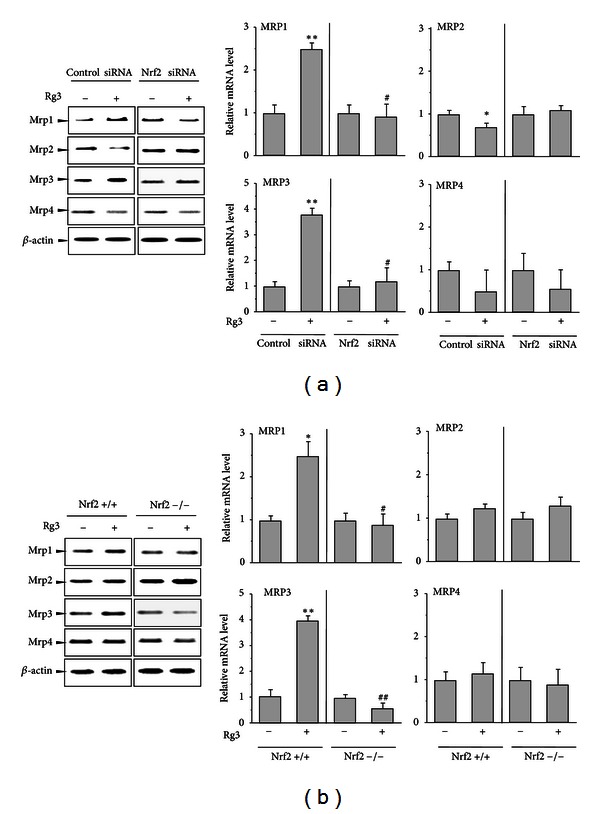
The effect of Nrf2 loss on the Mrp family gene expressions by Rg3. (a) H4IIE cells were transiently transfected with Nrf2-specific siRNA or control siRNA and subsequently treated with Rg3 (3 *μ*g/mL) or vehicle for 12 h. The representative blots of mRNA after Rg3 treatment were assessed by RT-PCR. *β*-actin was used as the loading control. (b) MEFs from wild-type (Nrf2+/+) or Nrf2-deficient mice (Nrf2−/−) were treated with Rg3 (3 *μ*g/mL) or vehicle for 12 h. The mRNA levels for the genes were analyzed as described above (a). The multiple analyses of 3 independent experiments were assessed by scanning densitometry. Each value represents the mean ± S.E. (**P < 0.01, *P < 0.05, significantly different from each control; ^##^ *P* < 0.01, ^#^ *P* < 0.05, significantly different from Rg3 in the control group).

Mrp family genes, which include Mrp1, Mrp2, Mrp3, and Mrp4, were reported to be Nrf2 target genes [18, 25]. We compared the Mrp family gene regulations by Rg3 in the Nrf2 knockdown or knockout with that in their respective controls. Rg3 increased Mrp1 mRNA levels by 2.5-fold in each control group in Figures 8(a) and 8(b), whereas the induction by Rg3 was not observed in either the Nrf2-siRNA transfected cells or the Nrf2-knockout MEF (Figure 8). Slight Mrp2 suppression by Rg3 was observed in control siRNA-transfected H4IIE cells, whereas Rg3 did not inhibit Mrp2 mRNA level in Nrf2 siRNA group, suggesting that Nrf2 may be a negative regulator of Rg3-mediated Mrp2 mRNA regulation (Figure 8(a)). Unexpectedly, Mrp2 mRNA by Rg3 was unchanged in Nrf2+/+ cells, and Mrp2 mRNA was enhanced by Rg3 in Nrf2−/− MEF (Figure 8(b)). This discrepancy of Mrp2 gene expression by Rg3 between H4IIE cells and MEF remains to be further assessed. Rg3 significantly increased Mrp3 mRNA in both of the control groups but not in either of the Nrf2-loss groups. Abolishment of the increased mRNA levels of Mrp1 and Mrp3 by Rg3 was evident in Nrf2-deficiency models, implying that Nrf2 is required for the induction of these genes by Rg3. On the other hand, there was no difference in the Mrp4 mRNA levels induced by Rg3 between the genotypes. These findings demonstrate that the Mrp mRNA levels that were differentially regulated by Rg3 were in part mediated by Nrf2.

## 4. Discussion

Previously, we found the beneficial effect of ginseng in response to hepatotoxicants via metabolic regulation involving suppression of CYP and induction of GSTA2 [3, 4]. Several studies have shown that ginsenosides have diverse pharmacological actions [5]. Among these constituents of ginseng, Rg3, which is produced from raw ginseng by the steaming process, is one of the red ginseng-specific components. Rg3 has been reported to exert various biological activities, including antiinflammatory, antiallergy, antitumor, and vascular relaxation [6–8]. Recently, work with Rg3 has been focused on the major stereoisomer 20(S)-Rg3, that is, converted from protopanaxadiol-type ginsenosides Rb1, Rb2, Rc, and Rd [26]. We demonstrated for the first time that 20(S)-Rg3, not Rg1 and Re, is an active ingredient responsible for the GSTA2 induction for hepatoprotection by Korean red ginseng [4]. 

The liver, the primary organ for the metabolism and detoxification of APAP, is the main target for its protection [4]. APAP is metabolized in the liver first by the CYP450 system and subsequently conjugated with glucuronide, sulfate, and GSH. CYP2E1-knockout mice were resistant to toxic doses of APAP, which suggested that CYP2E1 is involved in the formation of NAPQI [27]. We found that Korean red ginseng suppressed CYP2E1 expression, leading to retention of intact APAP [4]. NAPQI generated from APAP binds reduced GSH to form a stable conjugate and readily depletes intrahepatic GSH. The remaining NAPQI reacts with cellular proteins and causes formation of an adduct [10]. Here, the approach with NAPQI rather than APAP itself has allowed us to focus directly on the toxicity of the active metabolite. NAPQI at concentrations greater than 200 *μ*M resulted in significant increases in ALT and AST levels. This toxic range of NAPQI is consistent with a previous report in primary hepatocytes [10]. As shown in Figure 2, treatment of Rg3 followed by NAPQI attenuated the toxicity. Treatment with 1–50 *μ*g/mL Rg3 did not affect the viability of H4IIE cells (data not shown). These beneficial effects of Rg3, a natural component, give rise to therapeutic potential against hepatotoxicity of APAP.

NAPQI detoxification occurs primarily by GSH conjugation. In the clinic, N-acetylcysteine was reported to produce only a ~25% reduction in mortality from APAP insult [28]. Therefore, development has focused on potential alternative agents as therapeutic targets against APAP toxicity by restoring hepatic GSH content. A genome-wide association study suggested that the GSH pathway is partially associated with variations in NAPQI toxicity [29]. The current study showed that GSH was depleted, with subsequent increases in ALT and AST activities, when cells were exposed to NAPQI at doses of 200 *μ*M or higher. Rg3 significantly reversed the GSH depletion that was caused by a toxic dose of NAPQI. Interestingly, a low concentration of NAPQI (100 *μ*M) caused a mild increase in GSH level without producing toxicity (data not shown), which may have resulted from cellular defensive responses including upregulations of detoxifying genes to moderate oxidative stress [17]. However, a lethal dose of APAP fails to produce enough defense pathways to block the toxicity [4]. Collectively, this suggests that Rg3-mediated GSH repletion against NAPQI toxicity may be necessary for the hepatoprotection.

One possible explanation for the reversal of GSH depletion during NAPQI challenge is GSH biosynthesis [11]. As mentioned previously, GCL, also known as gamma-glutamylcysteine synthetase, is the rate-limiting enzyme of its synthesis. One study suggested that a cysteine prodrug is the recommended antidote for APAP, because it acts as a supplement to cysteine for GSH synthesis [30]. Although N-acetyl cysteine is still used today as the only approved drug to treat APAP toxicity, more direct mechanisms besides a GSH supply are required to protect cells from APAP insult [31]. Thus, the induction of both catalytic and regulatory GCL mRNA by Rg3 may facilitate GSH biosynthesis. The observed gene induction by Rg3 was accompanied by a dose-dependent increase in GSH levels. These results demonstrate that GCL-dependent GSH biosynthesis plays an important role in Rg3-mediated protection of NAPQI toxicity. 

The ARE region(s) in the 5′-flanking regions of many Nrf2 target genes including GSTA and NQO1 play a key role in gene regulation [32, 33]. We reported that Rg3 caused a great increase in luciferase activity in the ARE of the −1.65 kb GSTA2 promoter but not in the Nrf2-deleted promoter construct [4]. The significant enhancement of GSTA2 by Rg3 was attributable to convergence of 2 transcription factors, Nrf2 and C/EBP. As shown in Figure 4, Rg3 significantly increased the nuclear translocation of Nrf2 in a dose-dependent manner. The Nrf2 activation by a low concentration of Rg3 suggested that Nrf2 is likely to be the primary target of Rg3. Other studies such as the antiangiogenesis study used greater doses of Rg3 than what we used in this study [7]. We surmised that GCL gene induction by Rg3 is Nrf2 dependent as Nrf2 functions at the GSTA2 promoter [4]. The functional role of Nrf2 in the transactivation of these genes was confirmed in the Nrf2-deficient systems. Rg3 did not reverse the GSH depletion by NAPQI in the Nrf2 deficiency, supporting the previous studies findings that Nrf2 deficiency is highly susceptible to APAP-mediated toxicity *in vivo* [14]. In addition to its effects on the GSH biosynthesis genes, Rg3 also significantly induced antioxidant defense genes including NQO1, GSTA, thioredoxin reductase1 and superoxide dismutase 1, which are Nrf2-controlled target genes, implying that Rg3 leads to coordinated gene inductions that protects cells in response to oxidative insults (data not shown). These observations provide evidence that the activation of Nrf2 by Rg3 produces effects on multiple targets involved in protection against NAPQI toxicity. 

Recently, Mrp family genes were identified as Nrf2 target genes [18, 34]. We expected that Rg3 would upregulate Mrp family genes because Rg3 increased nuclear translocation of Nrf2 with transactivation of ARE. Indeed, Rg3 significantly increased mRNA levels of Mrp1 and Mrp3. Mrp1 contributes to Nrf2-dependent GCLC upregulation [35]. Interestingly, Rg3 suppressed Mrp2 mRNA levels. We also found that Korean red ginseng inhibited Mrp2 mRNA levels and protein expression (data not shown). Some research has demonstrated that other regulatory mechanisms (other nuclear receptors including PXR, FXR and CAR) might control Mrp2 gene expression, but only localized to the canalicular membrane [36]. Rg3 was reported to have mild glucocorticoid- and estrogen-like activities [5, 8]. Mrp2 suppression by Rg3 seems to be involved in other nuclear receptors for regulation, which are currently unknown. The MEF from wild-type mice showed that Rg3 induced mRNA levels of Mrp1 and Mrp3, which are consistent with effects in H4IIE cells. Unexpectedly, the decline of Mrp2 by Rg3 was not observed in the MEF cells. We assumed that this discrepancy of Mrp2 mRNA between H4IIE cells and embryonic fibroblasts might be due to their different machinery. The changes in mRNA levels of Mrp family genes by Rg3 were accompanied by changes in the protein expressions (data not shown).

The Nrf2 knockdown abrogated Rg3-induced Mrp1/Mrp3 gene induction and reversed Rg3-mediated Mrp2 suppression. Mrp2 mRNA by Rg3 was noticeably enhanced in Nrf2-knockout MEF. The role of Nrf2 in Mrp2 is still controversial. Two ARE sequences in the promoter region of the Mrp2 exist, and Nrf2 activators generated coinduction of Mrp and GCLC gene [18]. Conversely, the distal ARE sequence in Mrp2 most likely has a disposition for Maf homodimerization, which potentially negatively regulates Mrp2 [37]. A previous report demonstrated that Nrf2 plays a pivotal role in the constitutive levels of Mrp1 and Mrp3 but not those of Mrp2, suggesting that Mrp2 regulation is distinct and that other mechanisms may be involved [17]. Additional study of the distinct Mrp2 gene expression of Rg3 and its underlying mechanism is required. 

Resistance to APAP toxicity was attributed to Mrp3 up-regulation by Nrf2, which accelerated NAPQI excretion by efflux of APAP conjugates into the blood and suppression of its enterohepatic recirculation [20]. Conversely, low expression of Mrp3 in Nrf2 knockout decreased efflux of APAP conjugates [14, 18]. Intriguingly, Mrp2 in the canalicular transport system has an opposing effect in the APAP hepatic injury model compared with Mrp3 [14]. Apical Mrp2-deficient mice exhibited impaired excretion of conjugated APAP into the biliary tract with greater resistance to APAP toxicity than wild-type mice, and this excretion was accompanied by high levels of APAP-glucuronide in plasma [19, 38, 39]. Hence, the differential gene regulation on the Mrp family genes by Rg3 may be helpful to decrease bile excretion and facilitate basolateral excretion of NAPQI conjugates via Nrf2 activation.

In summary, our results demonstrated for the first time that Rg3 reduced NAPQI toxicity via repletion of GSH content in conjunction with GCLC and GCLM gene inductions. Rg3 differentially regulated Mrp transports, resulting in increased levels of Mrp1/Mrp3 and decreased levels of Mrp2. The Nrf2 pathway is required for the action of Rg3 on the Mrp transport and GSH biosynthesis, which likely contribute to the enhanced protection against NAPQI toxicity. Taken together, these results provide important information about the beneficial potential of Rg3 on the NAPQI-induced hepatotoxicity and coordinated regulation of detoxification and transport via Nrf2.

## Abbreviations

ALT: Alanine aminotransferase
APAP: Acetaminophen
ARE: Antioxidant response element
AST: Aspartate aminotransferase
GCLC: Glutamate cysteine ligase catalytic subunit
GCLM: Glutamate cysteine ligase modulatory subunit
GSH: Glutathione
Mrp: Multidrug resistance-associated protein
NAPQI: N-acetyl-p-benzoquinone imine
Nrf2: NF-E2-related factor 2
RT-PCR: Reverse transcription-polymerase chain reaction.
